# Editorial: Spatial immunology of tissue microenvironments

**DOI:** 10.3389/fimmu.2025.1584850

**Published:** 2025-03-18

**Authors:** Roslyn A. Kemp, Felix Marsh-Wakefield

**Affiliations:** ^1^ Microbiology and Immunology, University of Otago, Dunedin, New Zealand; ^2^ Liver Injury & Cancer Program, Centenary Institute, Sydney, NSW, Australia; ^3^ Human Immunology Laboratory, School of Medical Sciences, Faculty of Medicine and Health, The University of Sydney, Sydney, NSW, Australia

**Keywords:** spatial, human, lymph node, vasculature, tumor

While tools to study cells and molecules from tissue samples have provided important knowledge about the function of the immune system in health and disease, many have required the extraction of these cells from their normal environment. More recent technologies, in partnership with existing imaging platforms, have allowed the study of cells and molecules *in situ*. This approach allows the study of not just the heterogeneity of the immune response, but also the plasticity of immune cells. The latter is important because immune cell function is significantly influenced by the cellular neighbourhood – immune, stromal or tumour cells, secreted molecules, structural components such as vasculature, and the ability to make cell-cell contact. These new tools have allowed us to look at structures within the body, especially in human tissues, and have provided new biological information on immune function, as well as new ways to predict disease outcomes.

Many of these studies have been initially carried out in the context of the tumour microenvironment (TME). Cohn et al. review the importance of multiple factors in the TME and the tumour immune microenvironment (TIME) that have a known effect on patient outcome and which, therefore, need to be studied in their local surroundings. These authors highlight the role of cancer-derived factors, bacteria, and other immune cells in affecting patient outcome, and review how interactions between these factors and local immune cells are likely to direct these outcomes. These interactions also represent potential biomarkers of disease progression. Cohn et al. provide a comprehensive review of current imaging techniques, from traditional histochemistry-based approaches to sophisticated multi-parameter protein and RNA analyses. They highlight strengths and limitations of each technique and provide examples of key findings from each approach that have contributed to fundamental knowledge about the immune system in cancer. For any researcher considering spatial analysis of tissues, this paper provides a superb starting point to determine experimental and analytical approaches based on their specific research question.

Two examples of imaging technology and the importance of space and associated structures are provided by Moamin et al. and Femel et al. Moamin et al. use a simple multiplex immunohistochemistry (mIHC) approach to study immune cell phenotypes in triple negative breast cancer. Importantly, they study these phenotypes in the context of perivascular areas within the TME. They show that tumour-associated macrophages (TAMs) and T cells reside in different regions of the tumour. They extend their study to compare the location of cells in those patients treated with, and responsive to, neoadjuvant therapy. They identify an increase in stromal CD163+ TAMs in patients that responded to therapy, suggesting 1) a novel mechanism of cancer control and 2) a potential biomarker of tumour growth.

Femel et al., in a study of primary cutaneous melanoma patients, focus on identifying the different vessels that infiltrate the tumour. This information is essential when studying the TIME, since the lymphovasculature controls the movement of immune (and other) cells and therefore impacts immune surveillance and tumour escape. Importantly, they show that both the lymphovasculature and immune infiltrate are heterogeneous between patients, and that the localisation of different vessels rather than the density of the vessels was associated with the extent of the immune infiltrate. These researchers provide novel data on the types of vessels present in tumours and their importance in cancer outcomes. Furthermore, they provide a detailed methodology as a template for researchers to collaborate and create a high-resolution database of the vasculature of multiple cancer types.

While Moamin et al. and Femel et al. demonstrate the user of spatial analysis in tissues applied to cancer patients, Wang et al. and Khaba et al. provide new imaging-related resources for researchers. Wang et al. identify the potential for histological and spatial data and deep learning models to support our understanding of the TIME. Using a cohort of lung cancer samples, they developed a mIHC histopathological image classification dataset, validated against publicly available datasets. They tested two models to benchmark the classification and determined that transformer models were superior to convolutional neural network models in quantifying immune infiltrates and showing prognostic significance for patients. This now a publicly available dataset for researchers studying image quantification models as well as lung cancer outcomes.

Finally, Khaba et al. explore the feasibility of using whole lymph nodes as a source of information in HIV infected people. The rationale of the technique is that infections occur in tissues, not blood, and thus assays of blood provide only limited information about the biology of a disease. In a superbly designed study that integrated pathologists, patients and their communities, advocacy groups, bioethics specialists, and scientists, they showed that lymph node excision can be performed safely and reliably in the clinic. The main advantage of this approach over blood was the significantly higher yield of cells for study. This research provides a framework and protocol to investigate the lymph node as a source of information about immunity to disease.

Collectively, these five articles demonstrate the importance of including spatial information from tissues when studying health and disease, particularly in humans. They demonstrate examples of how imaging approaches can elucidate new mechanisms of immune function and cell interactions. Each study also highlights how analysing whole tissues can be used to better influence patient outcomes, which is the ultimate goal of many immunologists.

